# *Vibrio cholerae* Transmits Through Water Among the Household Contacts of Cholera Patients in Cholera Endemic Coastal Villages of Bangladesh, 2015–2016 (CHoBI7 Trial)

**DOI:** 10.3389/fpubh.2018.00238

**Published:** 2018-08-30

**Authors:** Zillur Rahman, Md. Anisur Rahman, Mahamud-ur Rashid, Shirajum Monira, Fatema-Tuz Johura, Munshi Mustafiz, Sazzadul I. Bhuyian, Fatema Zohura, Tahmina Parvin, Khaled Hasan, K. M. Saif-Ur-Rahman, Nazneen N. Islam, David A. Sack, Christine M. George, Munirul Alam

**Affiliations:** ^1^Infectious Diseases Division, International Center for Diarrhoeal Disease Research, Dhaka, Bangladesh; ^2^Department of Biotechnology and Genetic Engineering, Noakhali Science and Technology University, Noakhali, Bangladesh; ^3^Department of International Health, Johns Hopkins Bloomberg School of Public Health Baltimore, Baltimore, MD, United States; ^4^Department of Genetic Engineering and Biotechnology, University of Chittagong, Chittagong, Bangladesh

**Keywords:** cholera, *Vibrio cholerae*, household contacts, water, clonal transmission, PFGE, Bangladesh

## Abstract

Recurrent cholera causes significant morbidity and mortality in cholera endemic estuarine areas of Bangladesh. There have been limited studies to investigate the transmission patterns of *V. cholerae* associated with cholera in Bangladesh. In this study, we characterized *V. cholerae* serogroup O1 isolated from 30 cholera patients, 76 household contacts, 119 stored drinking water samples, and 119 water source samples in Bakerganj and Mathbaria, two cholera endemic coastal regions in Bangladesh. Results of phenotypic and molecular characterization of *V. cholerae* isolates (*n* = 56) confirmed them to be toxigenic belonging to serogroup O1 biotype El Tor (ET), and possessing cholera toxin of the classical biotype (altered ET). Molecular fingerprinting of the *V. cholerae* O1 of clinical and water origins determined by PFGE of *Not*-I- digested genomic DNA showed them to be closely related, as the PFGE banding patterns were highly homogenous. Phylogenetic analysis using dendrogram of cholera patients, household contacts, and household groundwater sources showed isolates within households to be clonally linked, suggesting water as an important vehicle of transmission of cholera in the coastal villages of Bangladesh. Transmission of toxigenic *V. cholerae* O1 through drinking water in cholera endemic rural settings underscores the urgent need for evidence based water, sanitation, and hygiene interventions promoting safe drinking water to prevent morbidity and mortality related to cholera and other enteric infections in Bangladesh.

## Introduction

Cholera is an acute dehydrating, potentially life-threatening diarrheal disease, transmitted through contaminated drinking water and poor WASH infrastructure and practices in low resource settings (1). Worldwide cholera remains a major public health problem can cause up to an estimated 4.0 million cases and 95,000 deaths annually (2).

Cholera is a severe form of acute diarrhea, caused by the gamma-proteobacterium *Vibrio cholerae*. Of the more than 200 serogroups of *V. cholerae*, only serogroups O1 and O139 which possess potent cholera toxin (CT) encoded by a filamentous prophage lysogenizing into the genome of the bacterium are responsible for the epidemic and pandemic cholera worldwide (3). Serogroup O1 has two biotypes: classical (CL) and El Tor (ET) which differ in major phenotypic and genetic traits (4).

*V. cholerae* O1classical biotype caused the sixth and presumably earlier pandemics out of seven cholera pandemics before being replaced with the ET biotype which has been responsible for the ongoing seventh pandemic since 1961 (4, 5). The El Tor biotype strains have undergone genetic changes such as a new hybrid El Tor carrying the classical biotype CT (6). These El Tor variant strains are referred to as “hyper-virulent” owing to their ability to produce more cholera toxin, greater spreading ability during epidemics, and increased competitive fitness for colonization than many ET isolates (7–9).

The actual mode of transmission of *V. cholerae* among cholera cases remains debated. Some study suggests that *V. cholerae* transmits fecal-orally through contaminated water (7, 10–12) following a “slow” human-to-aquatic environment-to-human pathway (13). However, a key element in transmission may be a recently recognized hyper infectious phase, which persists for hours after passage in diarrheal feces and can transmit rapidly among the household members of cholera patients following a faster “human-to-human” transmission route through fecal-oral contamination (13, 14). Cholera patients and the environmental reservoir can be considered as a potential source of outbreak but their respective relations to cholera transmission have been heavily disputed. Household members of cholera patients are at more than 100 times higher risk of a cholera infection than the general population (15). Furthermore, in spite of the substantial work done in this field, the exact source and mode of transmission for amplification of the disease to reach an epidemic status is still not completely elucidated.

Previous studies have shown the estuarine aquatic environment of the Bay of Bengal to be natural habitat for toxigenic *V. cholerae* (16–19). In these regions, the low salinity of rivers or shallow wells (averaging between 2.8 and 8.2 ppt) and temperatures between 26° and 35°C during the dry season (20) favors the growth and multiplication of *V. cholerae*. People living in this region are at high risk of cholera as salt intrusion (20) into the surface water bodies supports the growth and survival of toxigenic *V. cholerae*. The high population densities present an ideal environment for people to be infected with *V. cholerae* which causes deadly disease cholera (13, 18, 21). In addition, previous studies have found contaminated household water as an important risk factor for cholera among the household members and are likely attributed to sharing contaminated environmental sources or secondary transmission through poor hygiene practices (22–24).

In our recent cohort study investigating risk factors for cholera infections among 76 household contacts of cholera patients in the rural Bakerganj and Mathbaria, Bangladesh, we found that 37% of cholera patient households had a household member with a cholera infection by bacterial culture. Furthermore, 27% of stored household drinking water, and 13% of water sources in patient households had *V. cholerae*. In addition, household contacts with *V. cholerae* in their source water had a significantly higher odds of a symptomatic cholera infection 25. Rafique et al. (26), by analyzing *V. cholerae* O1 isolated from clinical cholera cases, their household contacts and drinking and water source samples as part of a randomized controlled trial of hospital based hand washing with soap and water treatment intervention [Cholera-Hospital-Based Intervention-for-7-Days (CHoBI7) Trial] (27) by pulsed-field gel electrophoresis (PFGE), showed water as the source of transmission of cholera among the study households in Dhaka city, Bangladesh. However, there are no studies, to our knowledge, that have investigated cholera transmission patterns in patient households in a rural setting, especially in coastal villages of Bangladesh which is important for identifying interventions to reduce cholera transmission. In the present study, we investigate person to person and environmental transmission routes for cholera infection among household contacts of cholera cases using pulsed-field gel electrophoresis (PFGE) of selected *V. cholerae* O1 isolated from coastal villages of Bangladesh namely Mathbaria and Bakerganj to identify the transmission routes that should be targeted in future interventions.

## Materials and methods

### Description of study sites

**Figure 1:**
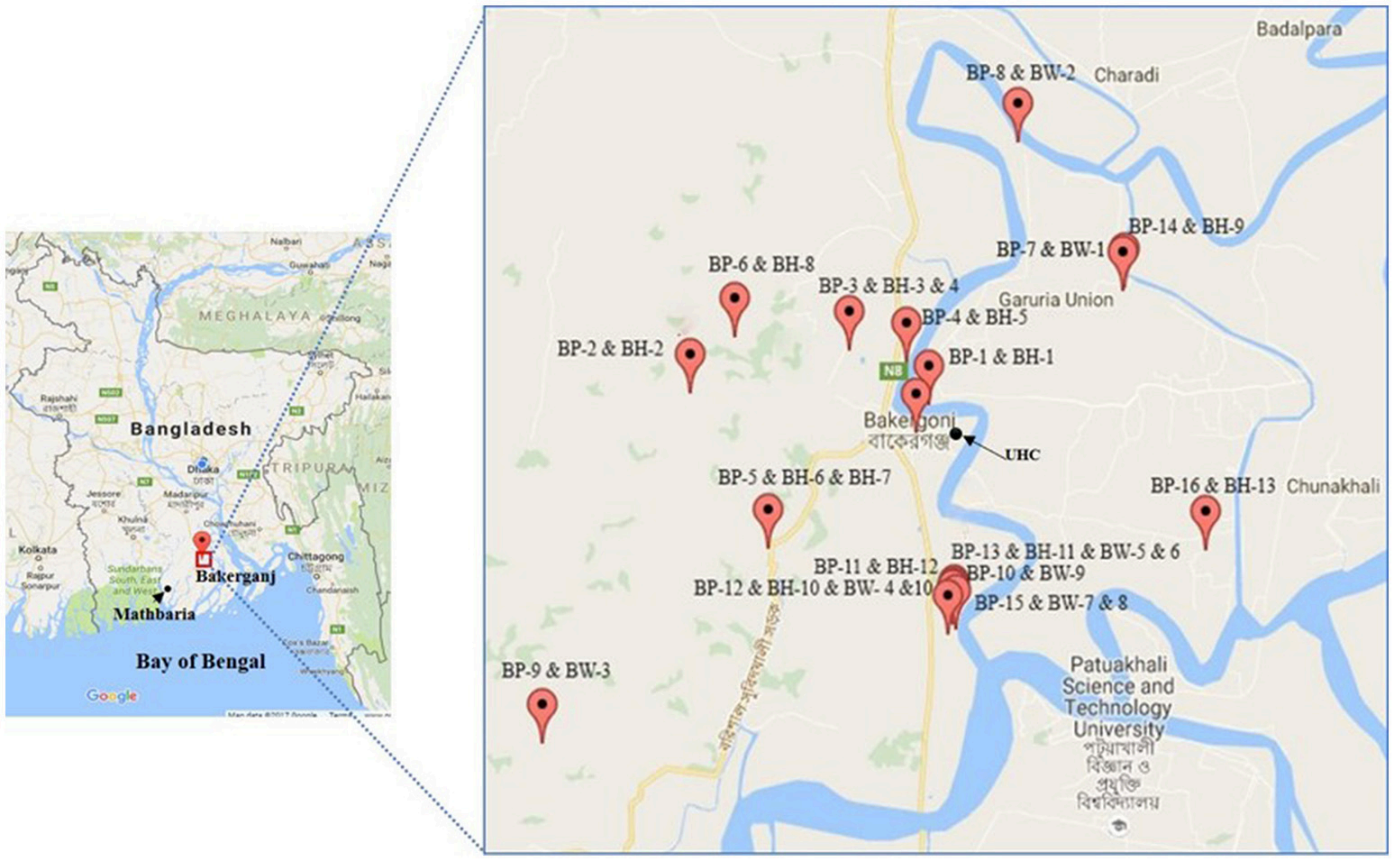
Map of Bakerganj with exact GPS (global positioning system) point for the location of households of cholera patients (BP, Bakerganj patient; BH, Bakerganj household contacts; BW, Bakerganj water; UHC, Upazila Health Complex). This map shows the distribution of representative (*n* = 16) households of cholera patients.

Our study was conducted in Bakerganj and Mathbaria, Bangladesh. Bakerganj is in the southern district of Barisal, about 70 km north of the coast of the Bay of Bengal and approximately 300 km southwest of Dhaka, the capital city of Bangladesh (Figure 1). Additional details on this study site are published in Alam et al. (16). Previous studies have shown significant correlations between the ecology and epidemiology of *V. cholerae* and selected environmental parameters in Bakerganj (21, 28).

**Figure 2:**
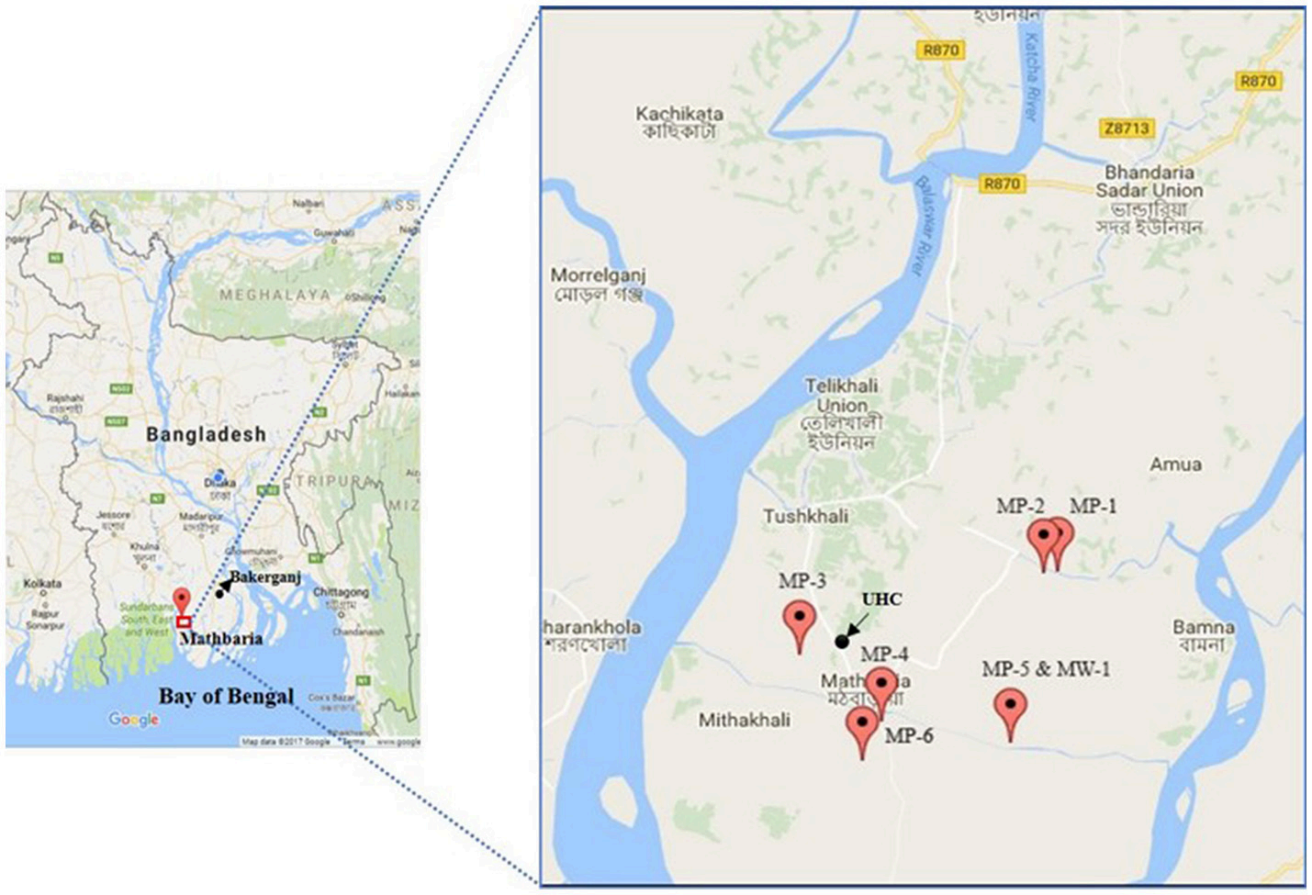
Map of Mathbaria with exact GPS (global positioning system) point for the location of households of cholera patients (MP, Mathbaria patient; MW, Mathbaria household; UHC, Upazila Health Complex). This map shows the distribution of representative (*n* = 6) households of cholera patients.

The second study site is Mathbaria, which is located adjacent to the Bay of Bengal, in close proximity to the Sunderban mangrove forest and approximately 400 km southwest of Dhaka (Figure 2). The major river, Baleshwar, flows along the western boundary of Mathbaria, next to a tropical mangrove forest of the Sundarbans, the temporary island system of that part of the Bay of Bengal. A previous study found the presence of toxigenic *V. cholerae* in the aquatic environment of Mathbaria which has been responsible for the endemicity of cholera in this area (17).

### Research procedures

#### Enrollment

Suspected cholera patients of any age and both sexes were recruited from hospitals in Bakerganj and Mathbaria. Suspected cholera patients (irrespective of age) were defined as anyone reporting diarrhea (3 or more loose stools within a 24-h period) and moderate to severe clinical dehydration (using WHO classification). All those individuals meeting these criteria who were admitted to Mathbaria and Bakerganj upazila government health complexes were screened for cholera using the Crystal VC Rapid Dipstick test. All individuals with a dipstick result positive for either *V. cholerae* O1 or O139, had their stool samples tested by bacterial culture for *V. cholerae*. A cholera patient was defined as an individual with diarrhea and a stool culture result positive for *V. cholerae*.

Once the index cholera patient was enrolled, their household contacts were invited to participate in the study. Household Contacts were defined as individuals sharing the same cooking pot with the cholera patients for the past 3 days. Household contacts of cholera patients residing were recruited and prospectively followed for 7 days after the presentation of the cholera patient at the local Government Upazila Health Complex (UHC). Eligible household contacts present in the health facility at the time of case enrollment were invited to participate. In addition, a household visit was made to recruit additional household contacts within 24–36 h of enrollment of the case. Household contacts were visited at 1, 3, 5, and 7 days for clinical and environmental surveillance.

Written informed consent was obtained from all study participants (household contacts and index cholera cases), this included adult participant (≥18 years of age) signing an informed consent and/or parental consent form and children between the ages of 12 and 17 years old signing an assent form. If a study participant could not read, the consent form was read to the participant and was asked to document their consent with an x in the presence of a witness. All study procedures were approved by the research Ethical Review Committee of the International Centre for Diarrhoeal Disease Research, Bangladesh (icddr,b) and IRB of the Johns Hopkins Bloomberg School of Public Health.

#### Clinical surveillance

For clinical surveillance, household contacts were asked if they had diarrhea or vomiting during the surveillance period, and fecal specimens were collected at each household visit to capture the majority of cholera infections that develop during the 7-day study period.

#### Environmental surveillance

Environmental surveillance was conducted during all household visits. Water samples were collected from the household's primary drinking water source and from drinking water stored in the home to measure *V. cholerae* by bacterial culture.

#### Sample collection and processing

Fecal specimens (stool) from index patients and their household contacts were collected aseptically and placed immediately in Cary-Blair media and transported to laboratory maintaining cold-chain. The water samples were collected using sterile 500 mL dark Nalgene bottles (Nalgene Nunc International, St. Louis, Mo.) and transported to the icddr,b laboratory putting in an insulated cool box (with ice packs). Stool samples from the cholera patients (index patient) were collected from Mathbaria and Bakerganj upazila government health complexes. Water samples were filtered through 0.22 μm filter papers and the membrane filters were then enriched in alkaline peptone water (APW) (pH 8.4) at 37°C for 4–6 h, and then cultured on selective media, as described previously (29). Stool samples were also subjected to APW enrichment and subsequent culture following same procedure. Clinical and water isolates were preserved for microbiological analysis.

#### Detection and characterization of V. cholerae

After culturing on selective media, isolation and identification of typical *V. cholerae* colonies were performed according to standard methods (30). The serogroups of the *V. cholerae* strains were determined serologically by a slide agglutination test using specific polyvalent antisera for *V. cholerae* O1 and O139. The serotypes of these strains were confirmed using serotype-specific monovalent Inaba and Ogawa antisera (29).

All phenotypically identified *V. cholerae* isolates were further confirmed by PCR targeting the *V. cholerae* species-specific gene *ompW* (31). Multiplex PCR assays were performed to identify genes encoding O1 (*rfb*O1) and O139 (*rfb*O139)-specific O biosynthetic genes, as well as the major virulence gene *ctxA* (32). To complement the biotype characterization by phenotypic traits, PCR assays were performed to detect the *tcpA* allele (CL and ET) (33), the type of the *rstR* gene, the presence of *rstC* gene encoding the phage transcriptional regulator (34), and the *rtxC* gene of RTX (repeat in toxin) (35) according to previously published methods. Double mismatch amplification mutation assay (DMAMA)-PCR was used to distinguish between the CL (*ctxB* genotype 1), ET (*ctxB* genotype 3) and Haitian types (*ctxB* genotype 7) of *ctxB* alleles by focusing on nucleotide positions 58 and 203 of the *ctxB* gene. DMAMA-PCR was performed in this study to detect the *ctxB* genotype using the primers and conditions described elsewhere (36).

#### Pulsed-field gel electrophoresis (PFGE)

The whole agarose-embedded genomic DNA for *V. cholerae* was prepared. PFGE was carried out with a contour-clamped homogeneous electrical field (CHEF-DR II) apparatus (Bio-Rad), according to procedures described elsewhere (37). Genomic DNAs of the test strains were digested by the *Not*I restriction enzyme (Gibco-BRL), and *Salmonella enterica* serovar Braenderup was digested by *Xba*I, with the fragments being used as molecular size markers. The restriction fragments were separated in 1% pulsed-field-certified agarose in 0.5X TBE (Tris/borate-EDTA) buffer. In the post electrophoresis gel treatment step, the gel was stained and de-stained. The DNA was visualized using a UV trans illuminator, and images were digitized by a 1D gel documentation system (Bio-Rad). The fingerprint pattern in the gel was analyzed using the Bionumeric software (Version 3.1). A dendrogram was constructed on the basis of banding similarity and dissimilarity using the Dice similarity coefficient and unweighted-pair group method (UPGMA) as recommended by the manufacturer.

## Results

### Occurrence and distribution of *V. cholerae* among study households

We performed genetic characterization of 56 *V. cholerae* O1 isolated from 30 cholera patients; 76 household contacts and 238 water samples (119 stored drinking water samples, and 119 water source samples) collected from 30 households. We have included the location of 22 representative households (16 in Bakerganj and 6 in Mathbaria) in Figures 1, 2. The map shows that majority of households with multiple *V. cholerae* infections in Bakerganj were located at the western and southern side of the Bighai river which is used for drinking, bathing and other household activities (e.g., washing kitchen utensils, vegetables) by nearby residents. In Mathbaria, all the households of cholera patients were located at the eastern side of Baleshwar river connected to the Bay of Bengal. From both maps, it was also found that households with multiple cholera infections were clustered around the Upazila Health Complexes (UHC) of Bakerganj and Mathbaria.

### Molecular characteristics of *V. cholerae*

All isolates were serologically confirmed to be *V. cholerae* O1, and all possessed “O” serogoup-specific gene *rfbO1* thereby complementing the serological results (Table 1). All *V. cholerae* strains belonged to serotype Ogawa; and all possessed the cholera toxin gene, *ctxA*. In addition, an ET biotype-specific toxin co-regulated pilus (*tcpA*^*ET*^), a phage transcription regulation gene (*rstR*^*ET*^), a phage transcription anti-repressor gene (*rstC*), and a repeat in toxin (*rtxC*) were found among in all *V. cholerae* O1 isolates, confirming the El Tor biotype traits. All *V. cholerae* strains possessed *ctxB* genotype 1, which is the classical type CT, confirming that the bacterium was El Tor but possessed classical biotype CT, characteristics of altered El Tor strains.

**Table 1:** Molecular characteristics of *V. cholerae* O1 isolated from cholera patients, their household contacts, stored drinking water and water sources.

**Case no**.	**Year of isolation**	**Location**		**Source**	***ctxA***	***ctxB* Genotype**	***tcpA* Type**	***rstR* Type**	***rstC***	***rtxC***	**Biotype**
		**Police station**	**Union**								
1	2015	Bakerganj	Rangosri	IP	+	Classical	El-Tor	El-Tor	+	+	Al-ET
				HC	+	Classical	El-Tor	El-Tor	+	+	Al-ET
				HC	+	Classical	El-Tor	El-Tor	+	+	Al-ET
2	2015	Bakerganj	Vorpasha	IP	+	Classical	El-Tor	El-Tor	+	+	Al-ET
3	2015	Bakerganj	Vorpasha	IP	+	Classical	El-Tor	El-Tor	+	+	Al-ET
4	2015	Bakerganj	Garolia	IP	+	Classical	El-Tor	El-Tor	+	+	Al-ET
				HC	+	Classical	El-Tor	El-Tor	+	+	Al-ET
5	2015	Bakerganj	Rongguchia	IP	+	Classical	El-Tor	El-Tor	+	+	Al-ET
				HC	+	Classical	El-Tor	El-Tor	+	+	Al-ET
6	2015	Bakerganj	Garolia	IP	+	Classical	El-Tor	El-Tor	+	+	Al-ET
				HC	+	Classical	El-Tor	El-Tor	+	+	Al-ET
7	2015	Bakerganj	Rangosri	IP	+	Classical	El-Tor	El-Tor	+	+	Al-ET
				HC	+	Classical	El-Tor	El-Tor	+	+	Al-ET
				HC	+	Classical	El-Tor	El-Tor	+	+	Al-ET
8	2015	Bakerganj	Rongoli	IP	+	Classical	El-Tor	El-Tor	+	+	Al-ET
				HC	+	Classical	El-Tor	El-Tor	+	+	Al-ET
9	2015	Bakerganj	Garolia	IP	+	Classical	El-Tor	El-Tor	+	+	Al-ET
				DW	+	Classical	El-Tor	El-Tor	+	+	Al-ET
10	2015	Mathbaria	Ghatichora	IP	+	Classical	El-Tor	El-Tor	+	+	Al-ET
11	2015	Mathbaria	Ghatichora	IP	+	Classical	El-Tor	El-Tor	+	+	Al-ET
12	2015	Mathbaria	Mirukhaly	IP	+	Classical	El-Tor	El-Tor	+	+	Al-ET
13	2015	Mathbaria	Mithakhali	IP	+	Classical	El-Tor	El-Tor	+	+	Al-ET
14	2015	Mathbaria	Tikikata	IP	+	Classical	El-Tor	El-Tor	+	+	Al-ET
15	2015	Mathbaria	Tikikata	IP	+	Classical	El-Tor	El-Tor	+	+	Al-ET
				DW	+	Classical	El-Tor	El-Tor	+	+	Al-ET
16	2015	Bakerganj	Rangosri	IP	+	Classical	El-Tor	El-Tor	+	+	Al-ET
17	2015	Bakerganj	Rangosri	IP	+	Classical	El-Tor	El-Tor	+	+	Al-ET
18	2015	Bakerganj	Rangosri	IP	+	Classical	El-Tor	El-Tor	+	+	Al-ET
19	2015	Bakerganj	Garolia	IP	+	Classical	El-Tor	El-Tor	+	+	Al-ET
				DW	+	Classical	El-Tor	El-Tor	+	+	Al-ET
20	2015	Bakerganj	Padri shibpur	IP	+	Classical	El-Tor	El-Tor	+	+	Al-ET
				DW	+	Classical	El-Tor	El-Tor	+	+	Al-ET
21	2015	Bakerganj	Vorpasha	IP	+	Classical	El-Tor	El-Tor	+	+	Al-ET
				HC	+	Classical	El-Tor	El-Tor	+	+	Al-ET
				DW	+	Classical	El-Tor	El-Tor	+	+	Al-ET
				SW	+	Classical	El-Tor	El-Tor	+	+	Al-ET
22	2015	Bakerganj	Vorpasha	IP	+	Classical	El-Tor	El-Tor	+	+	Al-ET
				SW	+	Classical	El-Tor	El-Tor	+	+	Al-ET
				DW	+	Classical	El-Tor	El-Tor	+	+	Al-ET
23	2015	Bakerganj	Garolia	IP	+	Classical	El-Tor	El-Tor	+	+	Al-ET
				HC	+	Classical	El-Tor	El-Tor	+	+	Al-ET
				HC	+	Classical	El-Tor	El-Tor	+	+	Al-ET
24	2015	Bakerganj	Vorpasha	IP	+	Classical	El-Tor	El-Tor	+	+	Al-ET
				HC	+	Classical	El-Tor	El-Tor	+	+	Al-ET
				SW	+	Classical	El-Tor	El-Tor	+	+	Al-ET
				DW	+	Classical	El-Tor	El-Tor	+	+	Al-ET
25	2015	Bakerganj	Vorpasha	IP	+	Classical	El-Tor	El-Tor	+	+	Al-ET
				HC	+	Classical	El-Tor	El-Tor	+	+	Al-ET
26	2015	Bakerganj	Vorpasha	IP	+	Classical	El-Tor	El-Tor	+	+	Al-ET
27	2015	Bakerganj	Vorpasha	IP	+	Classical	El-Tor	El-Tor	+	+	Al-ET
				SW	+	Classical	El-Tor	El-Tor	+	+	Al-ET
				DW	+	Classical	El-Tor	El-Tor	+	+	Al-ET
28	2015	Bakerganj	Vorpasha	IP	+	Classical	El-Tor	El-Tor	+	+	Al-ET
29	2016	Bakerganj	Koloskathi	IP	+	Classical	El-Tor	El-Tor	+	+	Al-ET
				HC	+	Classical	El-Tor	El-Tor	+	+	Al-ET
30	2016	Mathbaria	Tikikata	IP	+	Classical	El-Tor	El-Tor	+	+	Al-ET

*All isolates were confirmed as V. cholerae O1 Ogawa, by serological and molecular methods. IP, Index Patient (First Cholera patient from a family); HC, Household Contact (Family member of Cholera patient); SW, Drinking Water (Source); DW, Drinking Water (Household); ET, El-Tor; ctxA, Cholera Toxin A subunit; ctxB, Cholera Toxin B subunit; rstR, Repeat Sequence Transcription Repressor gene; tcpA, Toxin Co-regulated Pilus subunit A; rstC, Repeat Sequence Transcription Anti-repressor gene*.

### PFGE of *not*i-digested genomic DNA

PFGE analysis of *Not*-I- digested genomic DNA of 32 representative strains from hospitalized cholera patients, household contacts, stored drinking water and source water showed them to be closely related, as the PFGE banding patterns were highly homogenous.

**Figure 3:**
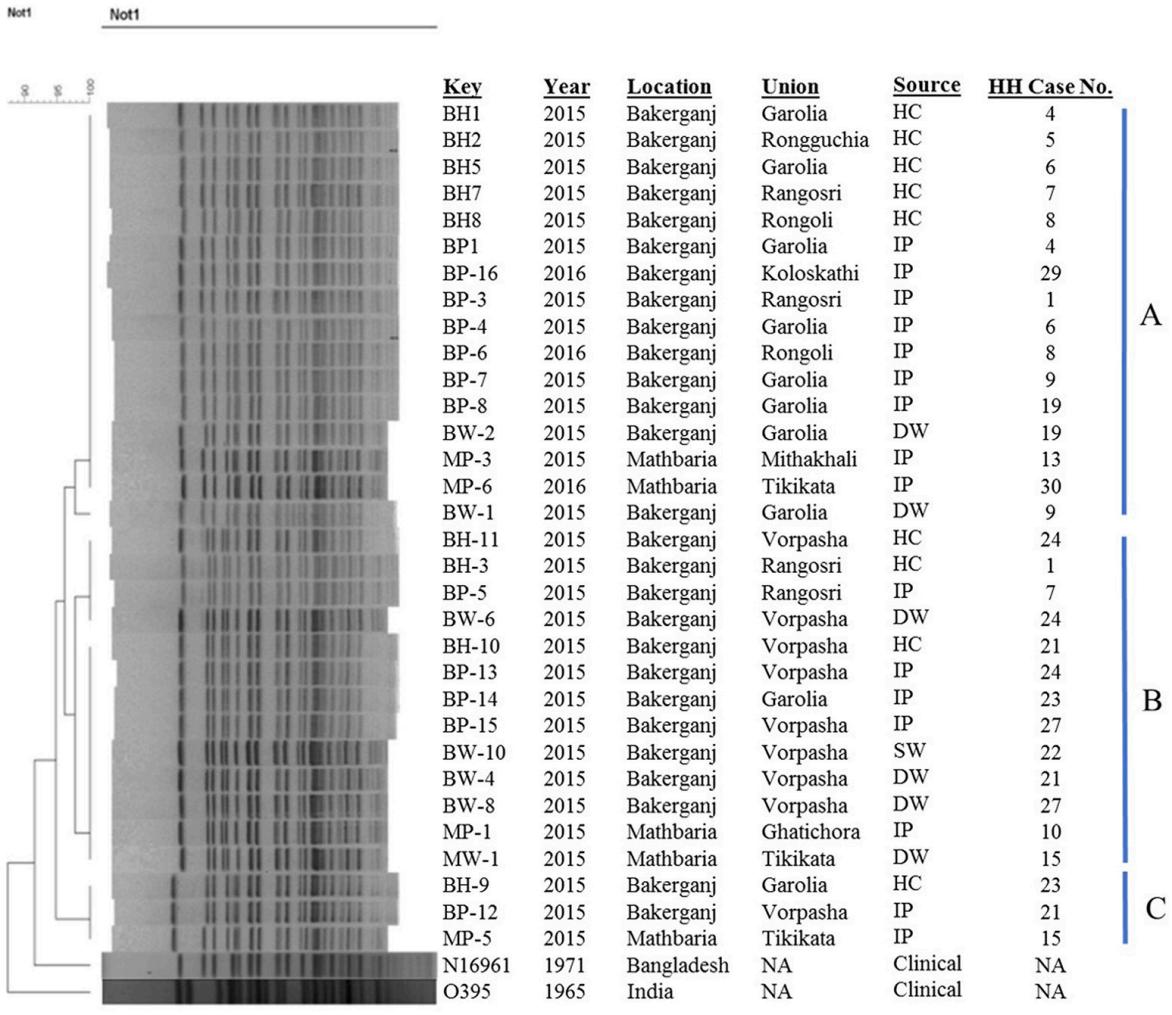
Genomic fingerprinting of *V. cholerae* O1 strains collected from environmental (water) and clinical (stool) sources of the household of index cholera patients. Thirty-Two *V. cholerae* O1 strains were isolated in Bakerganj and Mathbaria from hospitalized cholera patients (*n* = 16), their household contacts (*n* = 9), drinking water (*n* = 6), and source water (*n* = 1). The dendrogram was constructed by Dice similarity coefficient and mainstream hierarchical clustering (UPGMA) using PFGE images of Not1-digested genomic DNA. The scale bar at the top left indicates the similarity coefficient (range: 90–100%). IP, Index Patient; HC, Household contact; SW, Source Water; DW, Drinking Water; NA, Not Applicable.

The majority of the strains (*n* = 29) belonged to two major clusters A and B while rest of the strains (*n* = 3) showed discrete patterns and thus fell into a different minor cluster C (Figure 3). Cluster A, B, and C share 95–100% similarity co-efficient among them. Cluster A includes 16 isolates from index patients, household contacts, and drinking water sources from Bakerganj and Mathbaria of which 93.75% (15/16) isolates were found to be identical in their banding pattern. Cluster B comprised of 13 isolates of the same type and share 97% similarity. The minor cluster C comprised isolates from index patients and household contacts only. The N16961 (*V. cholerae* prototype El Tor) and O395 (*V. cholerae* prototype classical) reference strain showed significant differences with the altered El Tor strains analyzed in the present study.

## Discussion

Our phenotypic and genetic characterization data suggests that drinking water is an important transmission route for toxigenic *Vibrio cholerae* in cholera patient households in coastal villages in Mathbaria and Bakerganj, Bangladesh. This is consistent with our recent findings in Dhaka, Bangladesh showing a single *V. cholerae* ancestral clone and cholera transmission through drinking water in households of cholera patients (26). Most previous studies on household transmission of cholera in Bangladesh took place either in urban settings (15, 26, 38, 39) or in rural areas far away from coastal regions (22). This study is the first to report household transmission of toxigenic *V. cholerae* through drinking water in the cholera endemic coastal villages of Bakerganj and Mathbaria, Bangladesh.

Phenotypic and genetic characterizations of *V. cholerae* showed all belonged to serogroup O1, biotype El Tor (ET), and serotype Ogawa. All strains also had cholera toxin (CT) specific for classical biotype (40), which was replaced in the 1980s by ET biotype strains responsible for the ongoing 7th pandemic of cholera worldwide (41). Our results also confirmed all strains to be altered ET which has been associated with endemic and epidemic cholera in this region (16, 17, 40).

Historically, cholera has been endemic in coastal villages in the Bay of Bengal in both Bangladesh and India for centuries (2, 42). In the Ganges Delta of the Bay of Bengal recurrent cholera often turns into epidemics causing significant morbidity and mortality each year (16, 41). Previous studies have shown the aquatic environments of estuarine villages of Mathbaria to be an important reservoir for *V. cholerae* serogroups O1 and O139 which are responsible for epidemic cholera in this region (16). In coastal villages of Bangladesh, accessing drinking water remains a longstanding issue as the underground water is saline rich (16). As a result, coastal villages sometimes rely on surface water for drinking (16).

Mapping cholera patient households allowed us to investigate the spatial distribution of cholera patients in Bakerganj and Mathbaria. Through this analysis we found cholera patients to be highly clustered, with most residing near the Upazila Health Complexes (UHC) of Bakerganj and Mathbaria. Many cholera patient households also resided near ponds (water reservoirs) with small channels (canals) carrying water from the major rivers Bighai in Bakerganj and Baleshwar in Mathbaria. Both these rivers are in the tidal zone and are connected directly to seawater of the Bay of Bengal. Although the two study sites are geographically apart, both represent estuarine habitats favoring survival and multiplication of toxigenic *V. cholerae* (16, 17). Since only one household reported using pond water we suspect bathing in ponds and rivers and using pond water for household activities (e.g., washing kitchen utensils, vegetables) may also be a potential exposure route to *V. cholerae* that should be investigated in future studies.

Cholera transmission in coastal communities is of urgent public health importance because the warming climate globally could increase the number of coastal areas exposed to saline-rich water carrying *V. cholerae* and other pathogenic bacteria that flourish in elevated temperatures (43). Currently there is no standard of care for highly susceptible household contacts of cholera patients, instead the focus is on the use of oral rehydration solution (ORS) for the discharged cholera patient (42). George et al. (27) in a randomized control trial of the CHoBI7 intervention (Cholera Hospital-Based Intervention for 7 days), a hospital based handwashing with soap and water treatment intervention for cholera patient households, showed a 47% reduction in the incidence of cholera among household contacts of cholera patients with delivery of this intervention in Dhaka, Bangladesh (27). Therefore, the CHoBI7 intervention could present a promising approach to reduce cholera in rural coastal regions of Bangladesh and should be tested in future studies.

## Conclusion

Cholera remains a major public health problem worldwide especially in cholera endemic estuarine areas of Bangladesh where the estuarine habitat favors the survival and multiplication of toxigenic *V. cholerae*. This study provides evidence to support that toxigenic *V. cholerae* O1 is spread through contaminated drinking water among household contacts of cholera patients in the Bay of Bengal villages of Bangladesh. Transmission of toxigenic *V. cholerae* O1 through drinking water in cholera endemic rural settings underscores the urgent need for evidence based water, sanitation, and hygiene interventions promoting safe drinking water to prevent morbidity and mortality related to cholera and other enteric infections in Bangladesh.

## Author contributions

MA and CG are the Principal Investigators of the project and contributed to the design of the study, manuscript revision and final approval of the version to be published. SM is the functional Principal Investigator of the project and contributed to the design of the study, manuscript review and critical revision. ZR and MR designed and implemented the study. ZR performed the study in the laboratory and wrote the first draft of the manuscript. MAR, MM, and F-TJ performed the study in the laboratory and reviewed the manuscript. NI was involved in data analysis and reviewed the manuscript. KS-U-R oversaw the collection of data in the hospital/field and reviewed manuscript. FZ and TP were involved in data collection and contributed to manuscript writing. SB and KH were involved in database construction, data analysis and reviewed the manuscript. All authors read and approved the final manuscript. The authors have agreed to be accountable for all aspects of the work in ensuring that questions related to the accuracy or integrity of any part of the work were appropriately investigated and resolved.

### Conflict of interest statement

The authors declare that the research was conducted in the absence of any commercial or financial relationships that could be construed as a potential conflict of interest.
